# The long-term effect of COVID-19 infection on olfaction and taste; a prospective analysis

**DOI:** 10.1007/s00405-024-08827-2

**Published:** 2024-07-08

**Authors:** Tomer Boldes, Amit Ritter, Ethan Soudry, Dror Diker, Ella Reifen, Eyal Yosefof

**Affiliations:** 1 Department of Otorhinolaryngology and Head and Neck Surgery, Meir Medical Center, 59 Tchernichovsky St., 4428164 Kfar Saba, Israel; 2 Department of Otorhinolaryngology and Head and Neck Surgery, Rabin Medical Center-Beilinson Hospital, Petach Tikva, Israel; 3 Internal Medicine Department, Hasharon Hospital, Rabin Medical Center, Petach Tikva, Israel; 4 Faculty of Medicine, Tel Aviv University, Tel Aviv, Israel

**Keywords:** COVID-19, Anosmia, Parosmia, Ageusia, Smell, Chemosensory function

## Abstract

**Purpose:**

To estimate long-term prognosis of chemosensory dysfunctions among patients recovering from COVID-19 disease.

**Methods:**

Between April 2020 and July 2022, we conducted a prospective, observational study enrolling 48 patients who experienced smell and/or taste dysfunction during the acute-phase of COVID-19. Patients were evaluated for chemosensory function up to 24 months after disease onset.

**Results:**

During the acute-phase of COVID-19, 80% of patients reported anosmia, 15% hyposmia, 63% ageusia, and 33% hypogeusia. At two years’ follow-up, 53% still experienced smell impairment, and 42% suffered from taste impairment. Moreover, 63% of patients who reported parosmia remained with olfactory disturbance. Interestingly, we found a negative correlation between visual analogue scale scores for smell and taste impairments during the acute-phase of COVID-19 and the likelihood of long-term recovery.

**Conclusion:**

Our study sheds light on the natural history and long-term follow-up of chemosensory dysfunction in patients recovering from COVID-19 disease. Most patients who initially suffered from smell and/or taste disturbance did not reach full recovery after 2 years follow-up. The severity of impairment may serve as a prognostic indicator for full recovery.

## Introduction

The severe acute respiratory syndrome coronavirus-2 (SARS-CoV-2), also known as COVID-19, was initially reported in December 2019. In March 2020, the World Health Organization (WHO) announced COVID-19 as a pandemic. By April 2024, the WHO had registered over 775 million confirmed cases and over 7 million confirmed deaths worldwide due to COVID-19 [1].

The clinical manifestation of acute COVID-19 can range from asymptomatic to mild flu-like symptoms such as cough, myalgia, fatigue, and fever, and severe disease with respiratory distress and multi-organ failure [2]. Although this virus typically causes respiratory symptoms, it can also have neurological manifestations, including impairment of the sense of smell and taste. Moreover, smell and taste dysfunction are consistently reported among the most common complaints among patients [3]. Anosmia, the loss of smell, and ageusia, the loss of taste, have been proposed as important tools of measure for clinical triage [4]. The prevalence of chemosensory dysfunction was found to be similar in both vaccinated and unvaccinated, suggesting that symptoms such as anosmia and ageusia can potentially be valuable as diagnostic markers for suspecting COVID‐19, regardless of vaccination status [5].

Five variants of SARS-CoV-2, known as Variants of Concern (Alpha, Beta, Gamma, Delta, and Omicron), have been identified to date, and all of them have been associated with chemosensory dysfunction [6–10]. Since March 2023, these definitions have changed to “variants of interest” and “variants under monitoring”.

The persistence of symptoms after a COVID-19 infection, known as long-COVID, is a phenomenon in which symptoms continue for an extended period of time after the acute disease [11, 12]. The pathophysiology of long-COVID is not fully understood, but disturbances of taste and smell appear to be prominent symptoms [5, 11, 13]. Long-COVID can significantly impair the quality of life for some individuals and is considered an emerging pandemic concern [12].

Given the global prevalence of COVID-19, it is important to comprehend the long-term persistence of chemosensory dysfunctions. While anosmia and ageusia in COVID-19 patients have been the subject of numerous studies, most of these studies have only analyzed these symptoms up to a few months, and many have been retrospective. In light of this, our prospective study aims to investigate the long-term prognosis of chemosensory dysfunction in COVID-19 patients and to estimate the recovery rate among these patients from the early stages of the pandemic.

## Methods

### Ethical considerations

The study was approved by the Rabin Medical Center Institutional Review Board (IRB# RMC-20-0223). All patients signed their written informed consent to participate.

### Study design and participants

A prospective observational study from April 2020 to July 2022 consisted of 48 patients with confirmed COVID-19 via nasopharyngeal swab RT-PCR test. The study population consisted of adults aged 18 years or older who experienced either anosmia, hyposmia, ageusia, or hypogeusia during the acute-phase. Exclusion criteria included limited follow-up data, previous chemosensory dysfunction, pregnancy, and other neurological disorders.

### Initial assessment and data collection

Data was first collected through in-person medical history interviews and a thorough head and neck physical examination at our dedicated post-COVID-19 outpatient clinic. We collected demographic information and comprehensive clinical information on COVID-19 infection.

### Olfactory and gustatory evaluation

Smell and taste dysfunctions were evaluated throughout the follow-ups using the visual analog scale (VAS) [13, 14]. Patients were asked to rate their chemosensory impairment from 1 (anosmia/ageusia) to 10 (no impairment) prior to COVID-19 infection, during the disease, and throughout the follow-up.

### Follow-up timeline

In our research, we methodically structured the follow-up process into specific time frames, which we refer to as Checkpoint-1, Checkpoint-2, and Checkpoint-3.

At Checkpoint-1 in our post-COVID-19 outpatient clinic, patients underwent initial evaluations, on average two months post-infection (62 ± 34 days). During this stage, patients reported chemosensory impairments during the acute-phase of COVID-19. Subsequent follow-ups were performed through online questionnaires. Checkpoint-2 was scheduled for 9 months post-infection (mean 266 ± 72 days). At Checkpoint-3, we re-evaluated only patients who had not fully recovered from chemosensory dysfunction. This evaluation occurred 24 months after the initial infection (mean 678 ± 74 days).

### Statistical analysis

Statistical analyses were performed using SPSS, version 26. Continuous variables were compared using the Student T-test for normally distributed variables and Mann–Whitney U test for non-normally distributed variables and are presented as means ± standard deviations. Recovery rates based on VAS scores were compared using Fisher’s exact test. Categorical variables are presented as n (%).

## Results

A total of 48 patients with COVID-19 infection and chemosensory dysfunction were initially evaluated. Eight patients were excluded due to incomplete follow-up or insufficient data. Of the remaining 40 patients, 19 were males (47.5%) and 21 were females (52.5%), with a mean age of 51 ± 12.6 years.

Demographic and clinical features of all patients included in the cohort are presented in Table 1. Fifteen patients (37.5%) required hospitalization, with a mean stay of 11 ± 12.1 days of hospitalization and a median of 6.5 days. Supportive oxygen therapy was administered to 2 patients, and one other patient required intubation owing to respiratory failure.

**Table 1:** Features of 40 patients with chemosensory dysfunction

Background features (n, %)	
Gender	
Male	19 (47.5%)
Female	21 (52.5%)
Age	51 ± 12.6 years
Hospitalization	15 (37.5%)
Hospitalization period	11 ± 12.1 days
Self-quarantine	25 (63%)
Duration of COVID-19 infection	23.9 ± 13.8 days
Symptoms (n, %)	
Smell impairment	38 (95%)
Taste impairment	38 (95%)
Malaise	20 (50%)
Fever	19 (47.5%)
Cough	18 (45%)
Headache	14 (35%)
Dyspnea	13 (32.5%)
Myalgia	13 (32.5%)
Rhinorrhea	9 (22.5%)
Chest pain	3 (7.5%)

Our study focused on patients who reported chemosensory dysfunction during the acute COVID-19 infection. Thirty-six patients (90%) indicated concurrent disturbances in both olfactory and gustatory functions, two patients (5%) indicated only olfactory disturbances, and two patients (5%) reported only gustatory impairment. Other common symptoms were malaise, fever, cough, and headaches.

### Evaluation of the olfactory impairment

Retrospective inquiries about smell impairment during the acute-phase of the disease and prior to infection were conducted among all patients (Table 2).

**Table 2:** Changes in olfactory function over time

Olfactory function	
Pre-COVID-19	9.87 ± 0.47 VAS score
Acute disease	1.58 ± 1.62 VAS score
Anosmia	32 of 40 subjects (40%)
Hyposmia	6 of 40 subjects (15%)
Parosmia	8 of 40 subjects (20%)
Checkpoint-1 (2 months)	7.03 ± 3.15 VAS score
Checkpoint-2 (9 months)	7.91 ± 2.63 VAS score
Checkpoint-3 (24 months)	8.43 ± 2.58 VAS score
Improvement	33 of 36 subjects (92%)
Full recovery	17 of 36 subjects (47%)

**Fig. 1:**
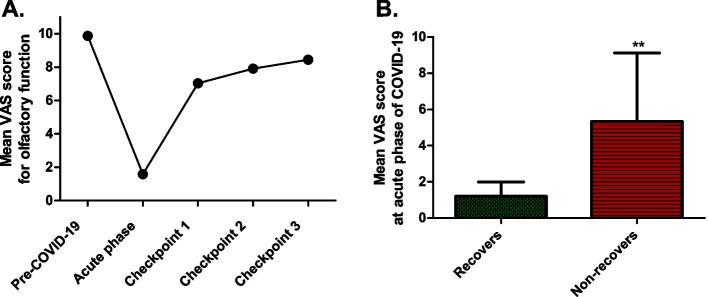
Subjective assessment of olfactory function. **A** Longitudinal changes in the mean VAS for olfactory function. **B** Initial mean VAS during acute COVID-19, comparing ‘Recovers’ and ‘Non-recovers’ groups, significant differences noted (p < 0.01)

The mean VAS score for olfactory function prior to the disease was 9.87 ± 0.47, while the mean score during the acute-phase was 1.58 ± 1.62 (Fig. 1a). Anosmia was reported in 80% of cases (32/40), while hyposmia was reported in 15% of cases (6/40). Only two patients did not experience smell but merely taste impairment (5%).

It is worth mentioning that in 47% of cases (18/38), smell impairment was the initial symptom of COVID-19 infection. However, in 13% (5/38), smell dysfunction appeared more than 7 days after the onset of other symptoms. In 47% of cases, the onset of smell dysfunction was abrupt (18/38).

The median duration until improvement in smell function was 10 days (ranging from 3 to 180 days). During the follow-up period, the mean VAS score for smell was 7.03 ± 3.15 at the first follow-up meeting and 7.91 ± 2.63 at Checkpoint-2 (Fig. 1a). After 12 months, two patients were lost to follow-up. The mean VAS score at Checkpoint-3 was 8.43 ± 2.58 (Fig. 1a). While 92% of patients (33/36) demonstrated some level of recovery in their ability to smell, less than half (47%, 17/36) achieved full recovery to their baseline olfactory function by the end of the follow-up period.

Patients presenting with a VAS score ≥ 7 during the acute-phase of COVID-19, indicative of mild hyposmia, demonstrated a persistent olfactory disturbance. Meanwhile, those with a VAS score < 7, such as those with anosmia or severe hyposmia, largely exhibited promising signs of partial or total recovery (p < 0.01 Fisher’s exact test, two-tailed). Specifically, the mean VAS score during the acute-phase for patients who recovered was 1.21 ± 0.78, suggesting severe initial olfactory impairment. In contrast, the mean VAS score during the acute-phase for patients who showed no signs of recovery was 5.33 ± 3.79, indicating milder initial symptoms (p < 0.01, Mann–Whitney U test, two-tailed, Fig. 1b). Additionally, the group of patients who achieved complete recovery demonstrated the most substantial improvement across all follow-up periods.

Interestingly, 20% of patients (8/40) reported parosmia, a distortion or misinterpretation of the perception of smell. Of them only three (37.5%) had fully recovered by the end of the follow-up period.

### Evaluation of the gustatory impairment

Additionally, all patients were retrospectively surveyed about their taste impairment before COVID-19 infection and during the acute-phase (Table 3), with mean VAS of gustatory impairment 9.92 ± 0.36 and 2.42 ± 2.4, respectively.

**Table 3:** Changes in gustatory function over time

Gustatory function	
Pre-COVID-19	9.92 ± 0.36 VAS score
Acute disease	2.42 ± 2.4 VAS score
Ageusia	25 of 40 subjects (63%)
Hypoegusia	13 of 40 subjects (33%)
Dysagusia	6 of 40 subjects (15%)
Checkpoint-1 (2 months)	8.14 ± 2.61 VAS score
Checkpoint-2 (9 months)	8.33 ± 2.16 VAS score
Checkpoint-3 (24 months)	8.73 ± 2.64 VAS score
Improvement	33 of 38 subjects (87%)
Full recovery	22 of 38 subjects (58%)

Gustatory impairment was reported by 95% of patients (38/40), ageusia in 62.5% (25/40), and hypogeusia in 32.5% (13/40). In all cases, loss of taste emerged concurrently with the earliest symptoms of COVID-19 infection.

**Fig. 2:**
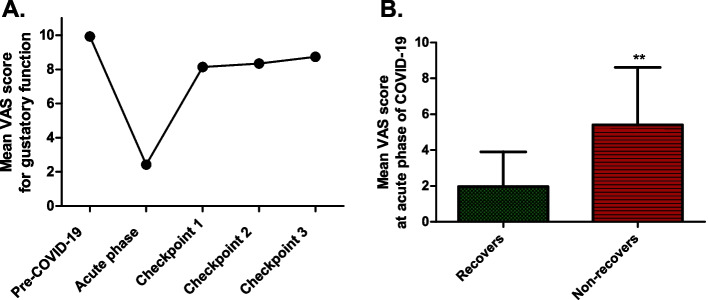
Subjective assessment of gustatory function. **A** Longitudinal changes in the mean VAS for gustatory function. **B** Initial mean VAS during acute COVID-19, comparing ‘Recovers’ and ‘Non-recovers’ groups, significant differences noted (p < 0.01)

The median time until improvement in gustatory function was 11 days (ranging from 3 to 45 days). The mean score for gustatory function consistently improved during the follow-up period, from 8.14 ± 2.61 at Checkpoint-1 to 8.33 ± 2.16 at Checkpoint-2, and 8.73 ± 2.64 at Checkpoint-3 (Fig. 2a). Most patients fully recovered (58%, 22/38 patients).

During the acute-phase of COVID-19, a VAS score ≥ 7, suggestive of mild hypogeusia, was notably linked to the prolonged persistence of taste impairment. In contrast, those with VAS scores < 7, representing ageusia or severe hypogeusia, tended to either partially or fully recover (p < 0.01 Fisher's exact test, two-tailed). Specifically, the mean VAS score during the acute-phase for patients who recovered was 1.97 ± 1.94, suggesting severe initial gustatory impairment. In contrast, the mean VAS score during the acute-phase for patients who showed no signs of recovery was 5.2 ± 3.56, indicating milder initial symptoms (p < 0.01, Mann–Whitney U test, two-tailed, Fig. 2b).

## Discussion

Our prospective study of 40 patients explored the long-term recovery from COVID-19-related chemosensory dysfunction. Initially, 90% (36 patients) experienced disturbances in both smell and taste. Although most patients showed some improvement during long-term follow-up, only half achieved complete recovery of both senses.

The impact of COVID-19 on smell and taste has been widely documented in the literature. A meta-analysis found that the overall prevalence rates for smell and taste impairments were 51% and 47.5%, respectively [15]. Additionally, a recent survey in the US showed that 60.5% of COVID-19 patients reported smell impairment, and 58% reported taste impairment [16].

Our findings indicate a median chemosensory improvement time of less than 2 weeks, aligning with studies that show significant improvements within weeks [14, 17], although many experience persistent symptoms. Extended studies confirm that a substantial proportion of patients, ranging from 27–37%, continue to suffer from chemosensory impairment up to 1 year [18–20]. Few studies have evaluated the prevalence of chemosensory dysfunction over 24 months. In the 2-year study by Boscolo-Rizzo et al., among 62 patients with mild COVID-19, 13 subjects (21%) showed partial recovery, and 5 subjects (8.1%) reported no improvement [21]. Their subsequent study reported a 20.5% prevalence of chemosensory dysfunction among patients with mild COVID-19 after 2 years (18/88 patients), with one additional patient recovering within a 3-year period (17/88 patients) [22]. Schambeck et al., study of 44 healthcare workers with mild or asymptomatic COVID-19 revealed that 11 participants (25%) experienced persistent disturbance in smell or taste after a median of 721 days. However, only 30 of 40 participants reported any change in taste or smell [23].

Importantly, while these studies are similar to ours, focusing on the long-term prognosis of chemosensory dysfunctions following COVID-19 recovery, they may suffer from selection bias due to concentrating on participants with mild symptoms. Additionally, in our study we estimate the long-term prognosis and the likelihood of recovery based on the initial severity of the self-reported impairments.

Hopkins et al. found that 93% of the subjects who reported parosmia continued to experience them after 6 months [24]. We observed a similar trend regarding parosmia: 63% of our patients who experienced parosmia continued to experience olfactory disturbances after 2-years. This finding is consistent with other studies that evaluate chemosensory dysfunction over periods ranging from 200 days up to 24 months [13, 23, 25], and it might suggest that parosmia could indicate an unfavorable prognosis.

Interestingly, our findings revealed a negative correlation between VAS scores for smell and taste impairment during the acute-phase of COVID-19 and long-term prognosis for recovery. Specifically, patients who presented with anosmia or severe hyposmia during the acute-phase displayed better recovery outcomes during follow-up than those with residual chemosensory function (VAS score ≥ 7). Similar to our findings, Iannuzzi et al. found a larger improvement in patients with anosmia compared with hyposmia subjects [26]. In contrast, Ohla et al. reported that patients who experienced a more significant reduction in their ability to smell were at a higher risk of long-term smell impairment, though the difference was numerically small [13].

Multiple theories explore the olfactory disturbance in COVID-19 without any universal consensus. The leading hypothesis suggests that disruptions arise from the olfactory epithelium, which is essential for smell perception and contains self-renewing cells [27]. Some studies highlight potential damage to non-neuronal cells [28], while others suggest direct damage to olfactory sensory neurons, altered neuron function due to immune responses, or other immune-mediated effects [29]. The impairment result from immune activation rather than the direct impact of the virus, possibly explaining the better recovery rates observed in our study for patients with acute-phase anosmia compared to those with milder hyposmia [29].

The different mechanisms of abrupt and long-COVID anosmia have yet to be deciphered. Biopsies from patients with persistent COVID-19 smell impairments showed ongoing inflammation in non-neural olfactory epithelial cells [28, 30], suggesting ongoing T-cell-mediated inflammation even after the virus had been cleared. Comparative analysis of long-COVID patients and infected hamsters revealed significant differences; human specimens showed prolonged T-cell infiltration, unlike the temporary immune response in hamsters, suggesting a distinct immunological response in long-COVID smell disorders. Yet, the samples often displayed an intact olfactory epithelium, challenging the idea that severe initial damage necessarily limits recovery [30].

Multiple studies suggest a possible correlation between a robust immune response and acute chemosensory impairment. Particularly, younger patients or those with fewer comorbidities seem more susceptible to acute-phase anosmia or ageusia [31–33]. Anosmia is also considered a favorable prognostic marker for milder COVID-19 progression [31, 34, 35].

Several studies have attempted to identify effective treatment interventions for chemosensory impairment. A Cochrane Living systematic review found no definitive results for interventions [36, 37]. Although there is currently no evidence supporting treatments to prevent persistent chemosensory dysfunctions, numerous trials are ongoing [36, 38].

Olfactory and gustatory functions are important in daily life, influencing diet, mate selection, and overall quality of life [39, 40]. Additionally, our sense of smell plays a crucial role in detecting life-threatening hazards such as toxic substances, fires, or gas leaks. Given that approximately 7% of COVID-19 patients might experience long-COVID anosmia [41, 42], 40% might have long-COVID hyposmia [41, 42], and 27% might suffer from hypogeusia [41], the global implications are enormous. With over 775 million reported cases to date, these chemosensory dysfunctions could potentially affect millions worldwide, leading to a substantial healthcare burden.

The present study has several limitations. First, our sample size was relatively small. Additionally, the evaluation of chemosensory dysfunction was based solely on subjective reports. These may not be as accurate as objective tests [43, 44] and could be susceptible to recall bias. However, some studies have suggested a positive correlation between subjective and objective methods [45].

## Conclusions

Since the start of the pandemic, extensive research has explored anosmia and ageusia in COVID-19 patients. However, most existing studies lack both prospective and long-term follow-up. Our research not only adds value to known literature but also sheds light on the natural history of chemosensory impairment through extended follow-up. After 2-years of follow-up, 53% of patients who initially presented with olfactory impairment during the acute-phase still reported smell disturbances. Additionally, by the end of the follow-up period, 42% of those who initially had gustatory impairment continued to experience taste disturbances.

Moreover, we identified a negative correlation between VAS scores for chemosensory impairment during the acute-phase of COVID-19 and the long-term prognosis for recovery. This leads to a central question: Can the severity of anosmia/ageusia during COVID-19 acute-phase predict long-COVID chemosensory disturbances? As new variants of COVID-19 continue to emerge, it is crucial to characterize and identify patients at high risk of enduring prolonged symptoms.
